# A health literacy intervention for registered nurses: Development and feasibility testing

**DOI:** 10.1016/j.ijnsa.2026.100503

**Published:** 2026-02-05

**Authors:** Malene Nerbøvik Stavdal, Marie Hamilton Larsen, Ingeborg Strømseng Sjetne, Astrid Klopstad Wahl, Caryl L. Gay, Monica Bukkøy Kjetland, Anners Lerdal, Christine Råheim Borge

**Affiliations:** a Department of Public Health and Interdisciplinary Health Sciences, University of Oslo, Norway; b Research Department, Lovisenberg Diaconal Hospital, Oslo, Norway; c Department of Postgraduate Studies, Lovisenberg Diaconal University College, Oslo, Norway; d Norwegian Institute of Public Health, Division for Health Services, Oslo, Norway; e Department of Family Health Care Nursing, University of California, San Francisco, USA

**Keywords:** Feasibility, Intervention, Health literacy, Health literacy sensitivity, Nursing

## Abstract

**Background:**

Health literacy is essential for improving long-term health outcomes, enhancing quality of care, and ensuring patient safety in healthcare systems. Thus, supporting registered nurses in applying health literacy in practice is essential. While health literacy research is expanding, limited attention has been paid to how healthcare personnel develop health literacy sensitivity (i.e., knowledge, skills, and attitudes healthcare personnel require to effectively meet patients’ health literacy needs).

**Objective:**

(1) Develop and assess the feasibility of a health literacy intervention for registered nurses working in a general hospital setting. (2) Explore potential changes in health literacy sensitivity, work-related factors, and mental well-being for registered nurses, following the intervention. (3) Evaluate registered nurses’ experiences with implementing the intervention.

**Design:**

Pre-post feasibility study employing qualitative and quantitative approaches.

**Setting:**

Medical ward, general hospital.

**Participants:**

*N*=32 registered nurses.

**Methods:**

An advisory panel, Magnet4Europe’s gap-analysis tool, and prior research were used to design the intervention. Health literacy sensitivity was measured using the Instrument of Health Literacy Competencies. Work-related factors and mental well-being were measured using four validated surveys. Quantitative data were analyzed using descriptive, non-parametric statistics. Registered nurses’ experiences with implementing the intervention were assessed through individual and group interviews and analyzed using thematic analysis.

**Results:**

The final intervention included a dedicated health literacy nurse trained to motivate and guide colleagues in improving patients’ health literacy. During a 6-month intervention period, registered nurses participated in internal health literacy education, project groups, morning briefings, and reflection groups. The intervention targeted all registered nurses (*N* = 32) working on a medical ward (i.e., cardiovascular, stroke, and geriatric conditions). The pre-post study showed statistically-significant improvements in 7 of the 9 domains measuring registered nurses’ health literacy sensitivity (pre *n* = 29, post *n* = 20). Additionally, teamwork scores between registered nurses and physicians showed a statistically-significant improvement. Qualitative (*n* = 9) analyses of registered nurses’ experiences of the intervention revealed three main themes: (1) “Strengthening health literacy through increased resources, embedded routines, and interdisciplinary collaboration,” (2) “Between support and strain: health literacy sensitivity as a professional challenge,” and (3) “Registered nurses’ increased awareness and understanding of health literacy sensitivity”.

**Conclusion:**

Post-intervention measures demonstrated a significant increase in registered nurses’ health literacy sensitivity through structured routines, interdisciplinary collaboration, access to resources, and support from a designated health literacy nurse. However, health literacy sensitivity remained a balance between rewarding engagement and burdensome responsibility.

**Trial Registration:**

Open Science Framework: https://osf.io/935vj/

**What is already known**

 

• Researchers have focused primarily on patients’ health literacy, with limited attention to healthcare personnel's health literacy sensitivity.
• Healthcare personnel often lack essential skills to address patients' health literacy challenges.
• Stressful work environments may impair healthcare personnel's ability to be sensitive to patients’ health literacy needs.

**What this paper adds**

 

• We found that the health literacy intervention improved seven of nine domains of health literacy sensitivity and strengthened interdisciplinary teamwork.
• We have raised awareness among registered nurses of the connection between health literacy sensitivity and patient safety.
• A designated health literacy nurse emerged as a key resource, motivating and guiding colleagues in prioritizing health literacy sensitivity by offering holistic insights into patients' needs.

Alt-text: Unlabelled box dummy alt text

## 1 Background

Health literacy sensitivity encompasses the knowledge, skills, and attitudes healthcare personnel require to effectively meet patients’ health literacy needs (Coleman et al., 2013; Sørensen et al., 2021; Karuranga et al., 2017) to “gain access to, understand, and use health-related information to promote and maintain good health” (Nutbeam, 1998, p. 357) for themselves, their families, and their communities (World Health Organization, n.d.). Individuals with health literacy difficulties often experience shame when interacting with healthcare personnel due to a lack of knowledge about their condition, communication barriers, limited involvement in decision-making, and difficulties navigating the healthcare system (Volandes and Paasche-Orlow, 2007; Sorensen and Brand, 2014; Le et al., 2021; Wynia and Osborn, 2010). Unfortunately, healthcare personnel do not always possess sufficient awareness or understanding of the challenges associated with health literacy, nor do they have the essential skills required to address them (Coleman et al., 2013; Karuranga et al., 2017).

Health literacy should be a fundamental element of the healthcare system, as it may influence long-term health outcomes, quality of care, and patient safety (Meggetto et al., 2020). Cocchieri et al. (2024) found that in hospital settings, low patient health literacy was associated with more nursing interventions, indicating greater treatment demands and a higher number of nursing diagnoses, potentially reflecting increased dependency. Thus, health organizations should provide guidance and support to healthcare personnel in understanding and utilizing the concept of health literacy in practice (Wilandika et al., 2023; Nutbeam, 1998). Registered nurses (RNs) play a pivotal role in healthcare, delivering professional nursing care and patient education. They are uniquely positioned to support patients in acquiring, understanding, and interpreting health information to manage conditions effectively (Wilandika et al., 2023). However, researchers have identified significant deficiencies in nursing students' knowledge of health literacy concepts and underscored the need for interventions to build critical competencies for better patient empowerment (DeBello and Wu, 2025; Anselmann et al., 2024). Furthermore, there are many hindrances to effective health literacy sensitivity, some of which stem from stressful work environments with heavy workloads and time constraints, which impact both healthcare personnel´s ability to deliver health literacy care and patients’ ability to engage with it (Cesar et al., 2022; Bruyneel et al., 2025).

Recently, researchers demonstrated a need for engaging RNs in health literacy interventions that did not increase their workload (Stavdal et al., 2025b; Stavdal et al., 2025a). For instance, we previously found that there was an urgent need for structured support and leadership engagement in promoting health literacy practices among RNs (Stavdal et al., 2025b). Additionally, healthcare personnel experiencing high levels of work engagement were more adept at addressing patients’ health literacy needs. We revealed that healthcare personnel with a heightened sensitivity to health literacy also reported better teamwork, more adequate staffing levels, and increased symptoms of depression (Stavdal et al., 2025b). This underscores the complex relationship between workplace dynamics and health literacy sensitivity among healthcare personnel (Stavdal et al., 2025a). RNs’ stressors at work have been found to be compounded by demanding patient care requirements and missed care, associated with reduced job satisfaction, emotional exhaustion, and higher turnover rates, which ultimately compromise patient safety and satisfaction (Bruyneel et al., 2025; Flowers et al., 2024; Karlsson et al., 2019). Conversely, positive work environments are characterized by safe staffing levels and more effective teamwork, which foster RNs' engagement, lead to lower burnout, and enhance job satisfaction, serving as a buffer against psychological distress (Carthon et al., 2019; Wang et al., 2019; Zhang et al., 2024).

Despite the growing body of health literacy research, the focus has predominantly been on patients, with comparatively less attention given to healthcare personnel’s health literacy sensitivity (Walters et al., 2020; Larrotta-Castillo et al., 2023; Marshall et al., 2025). Research is particularly needed on how health organizations can better support healthcare personnel in understanding and utilizing the concept of health literacy in practice (Wilandika et al., 2023). For instance, health organizations might use specific strategies to increase health literacy sensitivity, such as educating healthcare personnel, providing plain-language materials and navigation aids, and encouraging the use of communication techniques like the 'teach-back' (Wynia and Osborn, 2010; Brown et al., 2025).

Researchers who focused on patients' health literacy have found that using health literacy checklists during patient follow-up, in combination with building patient trust, may help patients receive and understand health information (McCaskill et al., 2024). Previous researchers conducting health literacy interventions for patients have demonstrated the impact of targeted strategies in enhancing patient knowledge, improving communication, raising awareness of health literacy issues, and fostering better patient self-management and trust in healthcare personnel (Brown et al., 2025; Andersen et al., 2022; Tsichla et al., 2025). However, we found no studies that specifically tested interventions aimed at improving RNs’ health literacy sensitivity while also considering their work-related factors and mental well-being.

### 1.1 Objective

The objective of this study was to:

(1) Develop and assess the feasibility of a health literacy intervention for RNs working in a general hospital setting.
(2) Explore potential changes in health literacy sensitivity, work-related factors, and mental well-being for RNs, following the intervention.
(3) Evaluate RNs’ experiences with implementing the intervention.

## 2 Materials and methods

### 2.1 Study design and setting

**Fig. 1:**
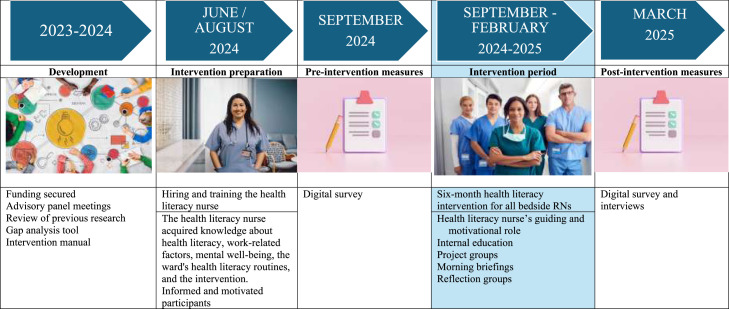
Development and feasibility testing of the health literacy intervention and timeline. Note: Registered nurse (RN).

This study was conducted in two phases at a medium-sized general hospital in Oslo, Norway. First, the intervention was developed, and then its feasibility (Bowen et al., 2009; Skivington et al., 2021) was evaluated over 6 months in a pre-post design with both qualitative and quantitative methods (Djojo et al., 2020). Fig. 1 describes these phases and the study timeline in more detail.

To ensure transparency and replicability, the study was reported in accordance with the guideline for reporting for intervention development studies (GUIDED) checklist (Duncan et al., 2020), and the template for intervention description and replication (TIDieR) checklist and guide (Hoffmann et al., 2014). This study is an independent satellite of the Magnet4Europe project, which aims to improve mental health and well-being in the healthcare workplace. The Magnet4Europe protocol is available elsewhere (Sermeus et al., 2022).

### 2.2 Developing the health literacy intervention

During intervention development, funding was secured, an advisory panel was established, and previous research was reviewed. The panel user representatives (a patient, a family member, and an RN), intervention ward management, and researchers collaboratively developed the intervention. Involving user representatives with target group experience in the conceptualization and design of the feasibility study was considered essential because of their unique insights (Bowen et al., 2009; Polit and Beck, 2012). Further, the collaborative planning with a diverse team, particularly in selecting the most appropriate research perspective and addressing practical obstacles, was fundamental to the study’s relevance and feasibility (Skivington et al., 2021).

The Magnet4Europe gap analysis tool was used to identify initiative-wide gaps and examples that guided systematic improvements and recognition pertinent to this study. Based on the results from our two previous studies (Stavdal et al., 2025a; Stavdal et al., 2025b), three key actions from the gap analysis tool were discussed to develop the health literacy intervention. These were: (1) providing nurses with opportunities to enhance their expertise in effectively educating patients and families, (2) fostering partnerships between nurses, patients, and families to co-create goals and plans for delivering patient-centered care, and (3) encouraging nurses to participate in interprofessional teams that implement and evaluate coordinated patient education initiatives (Magnet4Europe, 2020).

For instance, our local research indicated that healthcare personnel possessed limited motivation for adopting new health literacy tools or routines, perceiving them as difficult and increasing workloads; instead, they expressed a desire for having access to a dedicated resource person to consult and opportunities to share best practices for health literacy in patient follow-up (Stavdal et al., 2025b). Consequently, appointing a dedicated health literacy nurse to motivate and guide colleagues in enhancing health literacy sensitivity was proposed. This aligns with how nurses commonly learn, as they often prefer advice from colleagues as their primary source of information and heavily rely on their own and other professionals’ clinical experience (Fossum et al., 2022). It was suggested that the dedicated health literacy nurse should be an experienced RN working full-time in the intervention ward, appointed to a 20 % position to support colleagues in developing health literacy sensitivity work at a medical ward. A medical ward dedicated to nursing care (*N* = 32 RNs, *N* = 26 beds) for patients with cardiovascular disease, strokes, or geriatric conditions was recognized as the most suitable setting for implementing the health literacy intervention, given the patients' medical complexity. The ward management facilitated the selection of a health literacy nurse who was motivated, an effective communicator, eager to learn, proactive, and capable of guiding and leading others.

Training consisted of didactic, self-study, and clinical practice. Further, the employed health literacy nurse collaborated closely with the first author to gain a comprehensive understanding of her role and to plan details around the intervention. This included an overview of the project’s rationale and objectives, followed by an in-depth discussion of national health literacy challenges and strategies to address them. The training encompassed key topics, including health literacy concepts, work-related factors, mental well-being, the ward’s health literacy routines, and peer guidance. Nutbean’s (2020) three levels of health literacy—functional, interactive, and critical—were thoroughly examined to give the health literacy nurse an understanding that individuals’ health literacy is dynamic and changes over time. Additionally, the health literacy nurse explored the relationships between health literacy and various health-related outcomes, as well as the barriers faced by patients with low health literacy in navigating the healthcare system (Volandes and Paasche-Orlow, 2007), knowledge believed to be important when guiding and motivating colleagues to be health literacy sensitive. The critical role of healthcare personnel’s knowledge and skills in addressing patients’ health needs was emphasized, with particular attention to the unique contributions of RNs in promoting health literacy care (McCaskill et al., 2024). Significant effort was invested to ensure the health literacy nurse developed a comprehensive understanding of the intervention, which enabled her to contribute to its further development. Based on prior literature (Jensen et al., 2021; O’Hara et al., 2018; McCaskill et al., 2024; Brown et al., 2025), dedicated time was allocated for the nurse to master key communication techniques: Conversational Health Literacy Assessment Tool and teach-back. The health literacy nurse's mastery of these communication techniques was validated by experienced researchers and was important given her critical role in supervising RNs in their use. Additionally, strategies to guide and motivate colleagues were discussed, as well as the potential impact of the health literacy nurse’s new role on herself and her colleagues. Both Goeman et al. (2016) and Cawthon et al. (2014) have used similar strategies in their interventions, emphasising train-the-trainer models and the close clinical involvement of educators. All components from the development phase were incorporated into the intervention manual, which guided the intervention’s implementation.

### 2.3 Data collection

#### 2.3.1 Sample and recruitment

All clinical RNs working in the medical ward, treating patients with cardiovascular, stroke, or geriatric conditions, were invited and included. Information about the project was provided by the ward management and the first author through email and internal education. To assess the impacts of the intervention and RNs' experiences, a first survey was conducted before implementation, and a second survey, supplemented by qualitative interviews, was conducted after the implementation period (Moore et al., 2015).

#### 2.3.2 Pre- and post-intervention surveys

Validated instruments were used to collect survey data on key outcomes both before and after the intervention. The primary outcome was health literacy sensitivity, while secondary outcomes included work-related factors (teamwork and staffing) and mental well-being (work engagement, anxiety, and depression).

Primary outcome: Health literacy sensitivity

The Instrument of Health Literacy Competencies was used to explore participants’ health literacy sensitivity (Chang et al., 2017). The Norwegian version of this multidimensional instrument consists of 36 items (Borge et al., 2025) assessing nine domains: (1) Design teaching plan for low health literacy, (2) Simple and practical teaching, (3) Building a friendly environment, (4) Design of easy-to-use materials, (5) Life-oriented teaching, (6) Checking for understanding, (7) Encouraging questions, (8) Self-designed low-literacy materials to clients, and (9) Interdisciplinary collaboration. Domain mean scores range 1-5, with higher scores indicating greater health literacy sensitivity. Cronbach's alpha coefficients range from 0.56 in domain four to 0.89 in domain seven (Borge et al., 2025).

Secondary outcomes: Work-related factors

Secondary outcomes were assessed using relevant items from the Magnet4Europe survey (Sermeus et al., 2022). To assess Teamwork and Staffing, two items from the Practice Environment Scale were used: (1) A lot of teamwork between nurses and physicians, and (2) Enough staff to get the work done. Higher values indicate better teamwork/staffing. Lake et al. (2024) validated the Practice Environment Scale for use as single items.

Secondary outcomes: Mental well-being

Work engagement was measured using three items from the Utrecht Work Engagement Scale. Higher scores indicate higher work engagement. The Utrecht Work Engagement Scale is validated (Schaufeli et al., 2017). Anxiety was measured using the Generalized Anxiety Disorder-2 scale. Higher values indicate more frequent symptoms of anxiety. The Generalized Anxiety Disorder-2 is validated (Kroenke et al., 2007). Depression was measured using the Patient Health Questionnaire-2. Higher values indicate more frequent symptoms of depression. The Patient Health Questionnaire-2 is validated (Kroenke et al., 2003).

Participants' socio-demographic characteristics and occupational conditions were collected, including age, years of working as an RN, years worked in the intervention ward, patient group speciality (i.e., cardiology, stroke, geriatrics, or unknown), and familiarity with health literacy. To maintain confidentiality in the small sample, sex was not collected.

#### 2.3.3 Interviews

Individual and focus group interviews were conducted after the intervention to assess its feasibility.

1. An individual interview with the health literacy nurse was conducted on the last day of the intervention period.
2. Three focus group interviews were conducted with clinical RNs from the intervention ward within two weeks after the intervention period ended.

We invited participants through purposive sampling, selected specifically to ensure information-rich participants aligned with the study's objectives (Moore et al., 2015). To enhance group diversity, participants included RNs working across various patient groups, representing diverse ages, experiences, and levels of participation in the intervention project and reflection groups. The health literacy nurse was interviewed individually due to her unique role and to ensure that openness in the group was not influenced or constrained by her presence when discussing aspects related to her role.

A semi-structured interview guide was designed to explore participants' experiences with the intervention, including positive aspects, perceived barriers, suggestions for improvement, and the intervention’s impact on work-related factors and mental well-being. Before each interview, the research aim and the reasons for conducting the interviews were reviewed, and all participants were also asked to complete a brief survey regarding their socio-demographics and occupational characteristics. The interviews were audio-recorded and transcribed verbatim by the first author.

### 2.4 Data analysis

#### 2.4.1 Statistical analysis

Statistical analyses were conducted using Stata version 18.1. Descriptive statistics, including means and frequencies, were used to summarize RNs’ socio-demographic characteristics and occupational characteristics. Given that the data collected in the surveys before and after the intervention were not normally distributed, the Wilcoxon signed-rank test was employed to evaluate intra-individual changes between pre- and post-intervention measurements, and presented at the group level. The correlation coefficient effect size is reported as the rank-biserial correlation, and statistical significance was set at <0.05 (Kerby, 2014; Pallant, 2020).

#### 2.4.2 Analysis of the interviews

Following the six-phase guide by Braun and Clarke (2022), thematic analysis was used to analyze the interviews. Transcribed material was read, searching for meanings and patterns. The storage and retrieval software NVivo v12 (Jackson and Bazeley, 2019) was used for data management, assisting with the initial development of codes and themes. Two experienced qualitative researchers conducted the data analysis, and meetings were held to discuss patterns and themes, strengthening the methodological rigor. Further, five researchers discussed the clarity and completeness of these concepts before the overarching themes were identified, and disagreements regarding themes and subthemes were openly addressed. Presentation of themes are supported by key quotes (Table 1).

**Table 1:** Steps in reflexive thematic analysis by Braun and Clarke (Braun and Clarke, 2022).

**Phase:**	**Description of implementation:**
1. Familiarizing oneself with the dataset	An inductive approach was employed. The first author read the transcribed interviews twice and created notes and mind maps of interesting items and initial codes. This phase had no limitations, but the study's aim was considered as reflections began. The first and last authors discussed the mind maps and initial codes. Transcripts were sent back to a sample of participants for comments and corrections.
2. Coding	A list of initial codes from phase 1 constituted the first codebook. The first author coded the first interview using Nvivo software, identifying recurring patterns in the dataset and revising the initial codebook. The three other interviews were coded using the revised codebook. The first and last authors discussed the codebook and agreed on 23 codes to include. All interviews were coded a second time by the first author, but no new codes were added.
3. Generating initial themes	Codes were discussed and organized into three initial themes.
4. Developing and reviewing the themes	A thematic map was created to evaluate the themes, ensuring that each theme had a distinct focus and limitations, avoiding any overlap. A total of three overarching themes were identified.
5. Refining, defining, and naming the themes	A group of five authors discussed patterns and themes to ensure they accurately captured the primary content. Clarity and completeness were debated before the overarching themes were finalized and named. Themes were written using key quotes to support the data. Participants provided feedback on the findings.
6. Producing the report	The findings related to the study aim were presented and discussed in this paper, supported by key quotes.

### 2.5 Ethical considerations

The study was conducted in accordance with the Helsinki Declaration. It was approved by the South East Regional Ethics Committee for Health Research (REK, protocol 166980) and the hospital’s data protection official for research. Due to the intervention's impact on clinical practice, it was also presented and accepted by the hospital's administrators. Participants received oral and written study information and provided written consent before answering the online survey or participating in interviews.

## 3 Results

### 3.1 The final health literacy intervention

An experienced RN with special health literacy training served as the dedicated health literacy nurse, motivating and guiding colleagues in enhancing patients’ and families’ health literacy. Additionally, RNs participated in health literacy internal education, project groups, morning briefings, and reflection groups during the 6-month intervention period, which spanned from September 2024 to March 2025.

#### 3.1.1 The health literacy nurse’s motivating and guiding role

One day each week was designated as “Health Literacy Day”, when the health literacy nurse was available to motivate and guide colleagues in applying health literacy principles in practice. This motivational role was inspired by the intervention design of Djojo et al. (2020) and aimed to support RNs committed to improving health literacy follow-up for patients and their families. RNs were encouraged to utilize the health literacy nurse as a resource, remain open to feedback, and adjust their health literacy sensitivity. Priority was given to the health literacy needs of patients and families, as well as utilizing communication techniques. On each Health Literacy Day, the first author visited the intervention ward to support the health literacy nurse and to document shared observations and experiences for further analysis.

#### 3.1.2 RNs internal education

All RNs working in the intervention ward attended a 1-day internal education session at the beginning of the intervention, following the full-day format recommended by Rowlands and Hollar (2015). The first half included lectures covering such topics as the understanding of health literacy in relation to patients and conditions, health literacy sensitivity, work-related factors, mental well-being, intervention content, and its influence on RNs’ work. To enhance motivation, a user representative from the advisory panel shared his experiences with health literacy follow-up as a patient in the intervention ward. Additionally, the medical clinic director shared his views on the importance of health literacy sensitivity.

To ensure a common skill base, two communication techniques, the Conversational Health Literacy Assessment Tool (O’Hara et al., 2018) and teach-back (McCarthy et al., 2012), were introduced and practiced. Pocket-sized introduction cards for the Conversational Health Literacy Assessment Tool and teach-back were distributed to give RNs quick and easy access to key information in a fast-paced clinical environment. RNs then worked in pairs on practical exercises using the communication techniques in a fictitious patient follow-up scenario. Time was also allocated for willing RNs to complete the pre-intervention survey.

#### 3.1.3 RNs project groups to develop health literacy materials

During the intervention period, participants collaborated in project groups to explore self-selected health literacy topics and identified a need for practical health literacy materials on the ward, which previously lacked such resources. This approach aligned with Nutbeam's (1998) conceptualization of enabling in health promotion, which involves taking action in partnerships to empower patients with material resources to promote and protect their health. Drawing on Rowlands and Hallor’s (2015) findings that small group collaboration is an effective educational approach, four groups (15 RNs) developed tools tailored to patients and families. One group created an information booklet for patients and families with heart failure; another revised the information binder for families and patients affected by stroke. A third designed pocket-sized discharge checklists that combined practical measures with prompts for health literacy sensitivity, giving RNs quick access to key information to enhance efficiency. The fourth group produced an information brochure for families of patients experiencing delirium. The health literacy nurse and the first author facilitated and supported all groups.

#### 3.1.4 Morning briefings

Inspired by Cawthon et al. (2014), the health literacy nurse informed and repeated important health literacy topics and resources during morning briefings. These sessions also served as a platform for project group members to present the health literacy material that they had developed, fostering shared learning and engagement across the intervention ward.

#### 3.1.5 Reflection groups

The health literacy nurse also facilitated reflection groups during the intervention period, providing RNs with a structured space to share their experiences related to health literacy in clinical practice. In the second half of the intervention period, two groups were formed: one comprising three participants and the other 10, with attendance contingent upon clinical workload. Positive experiences from the first group helped recruit participants for the second.

### 3.2 Statistical results

Out of 32 eligible RNs, 29 completed the pre-intervention survey, resulting in a 91 % response rate. Table 2 shows the characteristics of the 29 participants who completed the pre-intervention survey and the nine who participated in the post-intervention interviews.

**Table 2:** Socio-demographic and occupational characteristics of participants completing the pre-intervention survey and post-intervention interviews.

**Characteristics:**	**Pre-intervention survey** **(*N=*29)**	**Post-intervention interviews (*N*=9)**
Age in years: Mean (SD) Minimum-Maximum	30 (8.7) 22-58	35 (11.8) 26-58
Years working as a registered nurse: Mean (SD) Minimum-Maximum	6 (7.5) 0-30	10 (11.6) 3-31
Years working in the intervention ward: Mean (SD) Minimum-Maximum	4 (4.1) 0-16	6 (4.1) 2-13
Full-time equivalent: Mean (SD) Minimum-Maximum	89 (15.6) 22-100	91 (8.7) 80-100
Patient group speciality, *n* (%): * Cardiology Stroke Geriatrics Unknown	11 (38%) 8 (27.5%) 8 (27.5%) 2 (7%)	5 (56%) 2 (22%) 2 (22%) 0
Familiar with the concept of health literacy from their education, *n* (%) Yes No Unknown	16 (55%) 6 (21%) 7 (24%)	4 (45%) 3 (33%) 2 (22%)

⁎ Registered nurses are assigned to one of three specialty groups (cardiology, stroke, or geriatrics) and work exclusively within their assigned patient group. Nurses working only nightshifts are an exception, working across all three groups. Note: standard deviation (SD), *N*= indicates the total number of participants.

One participant quit during the intervention, and 20 of the remaining 31 eligible completed the post-intervention survey, yielding a 65 % response rate. An attrition analysis comparing the pre-intervention characteristics of completers versus non-completers of post-intervention measures showed no statistically-significant differences.

A statistically-significant median increase from pre- to post-intervention was found for seven of the nine health literacy sensitivity domains, with effect sizes ranging from 0.35 to 0.47 (see Table 3). Of the secondary outcomes, teamwork between RNs and physicians was the only outcome that showed significant improvement from pre- to post-intervention, with an effect size of 0.48.

**Table 3:** Wilcoxon signed-rank test results for health literacy sensitivity, work-related factors, and mental well-being pre- and post-intervention (*n*=29 and *n*=20, respectively).

**Measures:**	**Pre-intervention (*N*=29)**		**Post-intervention (*N*=20)**		**W**	***p***	**Effect size *r***
	Median (IQR)	Min-Max	Median (IQR)	Min-Max			
Health literacy sensitivity: (scale: 1-5)							
**1. Design teaching plan for low health literacy**	**2.8 (2.3-3.2)**	**2-3.8**	**3.5 (3.3-3.7)**	**2.2-4.3**	**2.92**	0.002*	**0.46**
**2. Simple and practical teaching**	**3.6 (3.4-3.6)**	**2.8-4.2**	**3.9 (3.5-4)**	**3-4.4**	**2.49**	0.011*	**0.39**
3. Building a friendly environment	4.0 (3.6-4.3)	2.3-4.7	4.0 (3.7-4.3)	2.0-4.7	0.46	0.675	0.07
**4. Design of easy-to-use materials**	**3.7 (2.7-4.3)**	**2.3-4.7**	**4.0 (3.7-4.3)**	**2.3-4.7**	**2.70**	0.006*	**0.43**
5. Life-oriented teaching	4.0 (3.8-4)	2.3-4.8	4.0 (3.8-4.5)	3.5-5	1.26	0.215	0.02
**6. Checking for understanding**	**3.5 (3.3-4)**	**2-5**	**3.8 (3.8-4)**	**3.5-4.5**	**2.23**	0.024*	**0.35**
**7. Encouraging questions**	**3.0 (3-3.5)**	**1-4.8**	**3.5 (3.2-4)**	**1-5**	**2.25**	0.025*	**0.36**
**8. Self-designed low-literacy materials to clients**	**2.8 (2.5-3.3)**	**1.3-4**	**3.3 (2.9-3.8)**	**1.3-4.3**	**2.95**	0.002*	**0.47**
**9. Interdisciplinary collaboration**	**2.7 (2-3)**	**1-4**	**3.0 (2.5-3.5)**	**1.3-4.3**	**2.32**	0.019*	**0.37**
Work-related factors: (scale: 1-4)							
**Teamwork**	**2 (2-3)**	**1-4**	**3 (3-3)**	**3-4**	**3.05**	0.003*	**0.48**
Staffing	2 (2-3)	1-4	2 (2-3)	1-3	-0.09	0.100	0.01
Mental well-being:							
Work engagement (scale: 1-5)	3.3 (3-3.7)	2-4	3.5 (3.3-4)	2.3-5	1.44	0.157	0.23
Anxiety (scale: 0-3)	0.5 (0-1)	0-3	0.5 (0.25-1)	0-2	0.66	0.509	0.11
Depression (scale: 0-3)	0 (0-0.5)	0-2.5	0.5 (0-0.5)	0-1	0.77	0.442	0.12

IQR = interquartile range; Min-Max = minimum – maximum; W = Wilcoxon signed rank test; *r* = rank-biserial correlation coefficient, *N*= indicates the total number of participants.

Higher scores indicate greater health literacy sensitivity, better work-related factors, or better work engagement. High scores also indicate more anxiety/depression. Increased scores from pre- to post-intervention indicate improvement for all measures except anxiety and depression, where higher scores reflect increased symptom severity. The correlation coefficient effect size is reported as the rank-biserial correlation

⁎ Statistically-significant at the p<0.05 level. *P* reported is the exact *p*-value.

### 3.3 Results from the interviews

**Fig. 2:**
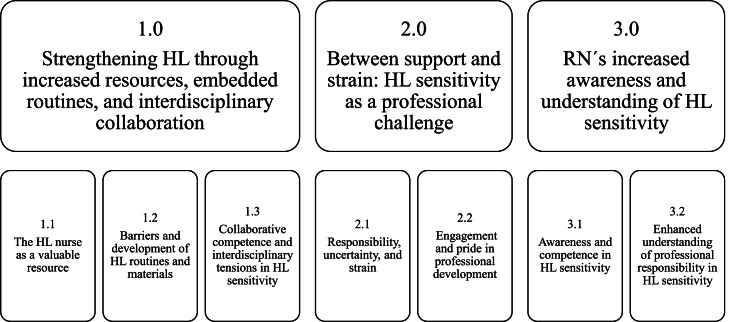
Overview of themes and subthemes from the qualitative data analysis. Notes: Three main themes were identified, each with two to three subthemes. Note: Health literacy (HL), Registered nurse (RN).

The perceptions of the clinical participants and the intervention’s health literacy nurse are presented below. The analysis identified three main themes, each with two to three subthemes, see Fig. 2.

#### 3.3.1 Theme 1.0: Strengthening health literacy through increased resources, embedded routines, and interdisciplinary collaboration

**Subtheme 1.1: The health literacy nurse as a valuable resource**

Some participants expressed that the health literacy nurse was an appreciated resource who provided guidance and support through a holistic understanding of patients’ health literacy needs, helping them successfully prioritize and organize health literacy follow-up.

“When I have a challenging case regarding patient discharge and collaboration with primary healthcare, it can be a bit difficult, but then suddenly the health literacy nurse has an overview from the morning and drops by to ask what health literacy measures are needed here? The health literacy nurse provides me with guidance and a review, which is quite nice, because she brings good insights and is a resource. " (Participant [P]:O)

Participants also wanted to preserve the health literacy nurse role and increase her involvement in following up on patients’ and families’ health literacy needs.

“In some cases, she is present, but I feel that most often she goes through the patient list and provides tips and support. However, it would be really great if she could physically accompany me to the patients. A bit of physical support, so that I could learn a little in that situation and not just talk about it beforehand.” (P: H).

**Subtheme 1.2: Barriers and development of health literacy routines and materials**

Producing their own health literacy materials seemed to strengthen participants’ health literacy sensitivity, and the material was considered important for enhancing the competence of all staff in the intervention ward. External health literacy materials have been organized throughout the intervention period; however, participants expressed that they still found it challenging to locate the necessary health literacy materials within the ward, and they have not become sufficiently familiar with the content of the materials.

“Important information like this is good to repeat orally as well, even if it's written down. I’ve talked to several colleagues about this, and it’s like, oh, do patients have to call the hospital themselves to schedule an appointment? I forgot to mention that, or I didn’t know. It’s written on the back. We need to understand the material and actually sit down with the patients to go through it with them. Because it’s helpful to receive it verbally in addition to the written brochure.” (P: M).

Information brochures were perceived as a practical support and were easier to use than information binders. However, some participants reported a lack of routines for using health literacy materials.

"I think it's a mix of being time-consuming and missing routines that hinder us from using those information binders. Since we haven’t established it as a routine, it quickly gets deprioritized. Perhaps informing patients should be given a higher priority." (P: O)

Participants viewed the interventions’ one-day internal education as providing a valuable introduction to communication techniques. Although some participants required regular training and reminders to use the techniques effectively, others perceived teach-back to be a useful clinical tool throughout the intervention period.

"For me, using teach-back to capture what the patient can understand and what they cannot understand makes me more conscious in the conversation; it helps me reflect on the information I give to patients." (P: A)

Additionally, it was expressed that during the intervention, participants began documenting patients’ health literacy needs and the health literacy measures they had completed in patient journals.

**Subtheme 1.3: Collaborative competence and interdisciplinary tensions in health literacy sensitivity**

Some participants valued colleagues' guidance and continuously used each other as sparring partners throughout the workday. The sense of unity and collaboration within the ward was appreciated by the participants as a valuable resource that enhanced their sensitivity to health literacy.

"We always discuss things with colleagues, just to get confirmation, if there's an important issue we’re wondering about, whether we are thinking correctly or not. I think we’re pretty good at being positive and using each other." (P: M)

Participants reported that patients can become frustrated when interdisciplinary health literacy follow-up is not systematized. While interdisciplinary teamwork can be viewed as a strength for health literacy sensitivity (Stavdal et al., 2025b), delegating responsibilities can diminish participants’ oversight and control.

“So, I start delegating right away, connecting with the physiotherapist. Fortunately, they came in this morning, so that was very nice. But then they come in with their own brochures, and it becomes a bit like, okay, do you have the opportunity to take that? Ultimately, I end up delegating the teaching responsibility to the physiotherapist. But I wonder, what they informed the patient about, and what do I need to pass on, because that part gets left hanging.” (P: O)

#### 3.3.2 Theme 2.0: **Between support and strain: Health literacy sensitivity as a professional challenge**

**Subtheme 2.1: Responsibility, uncertainty, and strain**

Participants found that health literacy sensitivity could be hindered by challenges such as time pressure, which could affect their conscience when they were prevented from fulfilling their responsibilities. The health literacy nurse found it difficult to guide and offer health literacy suggestions to colleagues during busy shifts, as they may be perceived as an additional responsibility. Some participants struggled to accept the health literacy nurse’s well-meaning suggestions, which sometimes made health literacy sensitivity feel more like an obligation than a resource.

“I've had a guiding role that I’ve found a bit challenging. I've supervised students, but being a colleague mentor has been very different. I think it's been a bit challenging during the shifts I've had as a health literacy nurse, knowing how it is out in the ward, how busy it is, how little time they have. And then I try to stop them on the way somewhere important to ask them if maybe this patient could be enrolled in patient heart school or maybe you've used teach-back today? I've felt a bit guilty because I feel like health literacy sensitivity has become an extra responsibility, even though it's part of their job. It feels like nagging, even though they are very motivated, to get them to spend more time on it." (P: D)

Furthermore, some participants also worried that they did not have enough knowledge to be truly health literacy sensitive. They pointed out that meeting patients’ and families’ information needs takes both time and resources. Simultaneously, the intervention’s educational and guiding focus contributed to a strengthened work environment by creating a positive atmosphere in the ward and generally reducing participants’ stress.

"When resources are invested in a health literacy nurse, I get the feeling that we aren’t as understaffed. That we prioritize essential academic content of the nursing profession, even if the ward is full. It contributes to a positive atmosphere. Yes, there's a bit of stress today, but we have someone focusing on professional content and health literacy, and she provides a different focus within the ward, and that has positively impacted stress and the atmosphere." (P: E)

**Subtheme 2.2: Engagement and pride in professional development**

The health literacy nurse was committed and excited about her role. Many participants experienced enthusiasm and motivation to participate in health literacy project groups and professional development, and they took pride in the health literacy materials they produced.

"There has been a lot of enthusiasm around those project groups; it was intense work, and people really enjoyed it. Something useful absolutely came out of it; something new was created in all the groups." (P: W)

#### 3.3.3 Theme 3.0: RNs’ increased awareness and understanding of health literacy sensitivity

**Subtheme 3.1: Awareness and competence in health literacy sensitivity**

There seemed to be a subtle shift in attitudes among participants during the intervention period. They acknowledged the importance of assessing patients’ health literacy levels, tailoring information, and recognizing literacy. However, they did not always attribute these changes to the intervention measures.

"No, I'm a bit unsure; I feel there haven’t been that many measures. When I think about this intervention, I mostly think about the health literacy nurse who has been there on Tuesdays. She has been available and very engaged, taking her role very seriously. She has made us aware of the patients' needs and asked us questions. I actually think that has been nice." (P: H)

Nevertheless, some participants became increasingly aware of how patients’, families’, and their own sense of safety connect to health literacy sensitivity. They have also increased their efforts to check the patient’s understanding and involve family members in health literacy measures.

"I often see that patients' health literacy reflects our health literacy as RNs; I'm quite aware of that. Patients who have an RN who provides a lot of information and checks that they understand it are much safer, as are those RNs." (P: D)

**Subtheme 3.2: Enhanced understanding of professional responsibility in health literacy sensitivity**

The participants have developed a stronger understanding of their role and responsibility in meeting patients’ and families’ health literacy needs throughout the intervention period. Participants also increasingly appreciated the health literacy nurse’s contribution and expressed that her mere presence raised awareness of the importance of health literacy sensitivity.

"The health literacy nurse is a great reassurance, that's for sure. She is very steady, so having her here has been a comfort. She is always available but has made it very clear that she is not here to take over for us; rather, she is here to support and assist us. So that has been very good." (P: W)

## 4 Discussion and conclusion

### 4.1 Discussion

We have presented the development and feasibility testing of a health literacy intervention for nurses to follow up on patients’ health literacy needs and to assess RNs’ experiences with the intervention. Additionally, we have explored potential changes in RNs’ health literacy sensitivity, work-related factors, and mental well-being. The quantitative and qualitative results indicate that the intervention seems to be feasible and helpful for participants’ health literacy sensitivity; both evaluations are positive.

In recent years, nurse-led health literacy interventions have gained increasing prominence, emphasizing their vital role in enhancing outcomes for both patients and caregivers. Larrotta-Castillo et al. (2023) reviewed health literacy interventions in hospital settings, identifying numerous strategies. However, they underscored the need for additional mechanisms to significantly improve health literacy for patients and caregivers. Their findings highlight the importance of all healthcare personnel, especially nurses, in implementing these interventions effectively (Larrotta-Castillo et al., 2023). Nurses are uniquely positioned to support patients in acquiring, understanding, and interpreting health information to manage illnesses effectively (Wilandika et al., 2023). Marshall et al. (2025) recently found that individual-focused health literacy interventions enhance health literacy-related outcomes, particularly among older adults. Despite these promising developments, systematic reviews tend to overlook work-related factors or the mental well-being of RNs (Marshall et al., 2025). Nevertheless, such review articles collectively reflect a rising trend in health literacy interventions and underscore the need for further research to optimize intervention strategies, while also addressing the support systems necessary for nurses to implement them in healthcare settings.

An important finding of the feasibility study of this health literacy intervention is that participants gained a better understanding of the importance of health literacy for patients and families, and increasing awareness of how patients’, families’, and their own sense of safety is connected to health literacy sensitivity. This outcome represents a notable advancement in understanding that patient safety cannot be guaranteed without addressing the negative perceptions associated with low health literacy (Loan et al., 2018). Patient health literacy is associated with nursing care complexity, as low health literacy reflects greater dependency and leads to more nursing interventions, thereby increasing treatment demands (Cocchieri et al., 2025). Furthermore, low health literacy contributes to adverse outcomes, such as increased clinical risks and higher resource use, including prolonged length of stay. Moreover, RNs play a pivotal role in enhancing patients’ understanding of their medical conditions and available treatment options (Loan et al., 2018; Wilandika et al., 2023). Through their observations, assessments, and deliberations, RNs can provide person-centered health literacy follow-up. Patients’ interactions with RNs represent important trusted relationships through which patients may be able to obtain and process health information (McCaskill et al., 2024). It is, therefore, essential that RNs also possess sufficient health literacy, as poor health literacy sensitivity among RNs can lead to distrust from patients and families (Djojo et al., 2020).

During the 6-month intervention, the role of participants in promoting optimal health outcomes gained greater emphasis. By informing and educating patients and families, RNs deliver critical information and equip them with the knowledge and health literacy skills necessary for effective health management (Wilandika et al., 2023). However, being health literacy sensitive can be perceived as both meaningful and burdensome. Our participants explained that health literacy sensitivity could be experienced as an added responsibility. Wittenberg et al. (2018) had similar findings and described that nurses view health literacy support as a core advocacy role that contributes to patient empowerment of health behaviors but can also be burdensome due to emotional and psychological demands.

The health literacy nurse emerged as a valued resource among colleagues, who took pride in helping participants increase their health literacy understanding, skills, and practice. Through a holistic understanding of patients' health literacy needs, the health literacy nurse provided guidance and support, enabling participants to successfully prioritize and organize health literacy follow-up. Participants appreciated this support from the health literacy nurse and their colleagues, often engaging in peer collaboration and acting as sparring partners for one another. However, one must be aware that introducing a health literacy nurse role may bring challenges, such as complicating existing workflows or adding a communication layer that requires careful coordination. This can unintentionally increase workloads or create ambiguity regarding roles and responsibilities within interdisciplinary teams. Researchers have highlighted that the successful integration of health literacy roles in healthcare settings requires strong leadership, strategic planning, clear role delineation, and active team engagement to prevent role confusion and workload issues (Pelizzari et al., 2025).

Participants experienced an increased sense of unity and collaboration within the ward, and their appreciation for interdisciplinarity grew throughout the intervention period, as it was seen as a valuable resource that enhanced their sensitivity to health literacy. Good nursing work environments are characterized by strong communication and effective teamwork with physicians (Carthon et al., 2019). Such collaborations are crucial for healthcare organizations to coordinate and integrate care (Trezona et al., 2018). Qualitative findings suggested that some participants perceived increased engagement and reduced stress following the intervention; however, these perceptions were not supported by statistically-significant changes in the quantitative measures.

Although participants sometimes experienced a troubled conscience when time pressures during busy shifts limited their ability to practice health literacy sensitivity, we found that the intervention increased their engagement and reduced work-related stress. By fostering RN job satisfaction and work engagement, healthcare organizations may reduce burnout, improve mental health, and empower nurses to deliver exceptional patient care (Green et al., 2020; Zhang et al., 2024; Moreno-Martínez and Sánchez-Martínez, 2025). Although an intervention requiring participants to alter their work habits might be expected to increase staffing needs or decrease their work engagement, that was not observed in this study. Instead, we found a statistically-significant improvement in teamwork scores following the intervention, with no detrimental effects on work-related factors or mental well-being.

Interviews indicated that participants were enthusiastic and motivated to engage in the intervention, particularly through participation in project groups focused on developing materials aimed at enhancing patients’ and families’ health literacy knowledge, while potentially also enhancing their own health literacy expertise through working with these materials. Working in small groups was described as an efficient educational approach, well-suited for healthcare personnel to develop health literacy measures that can meet the needs of patients and families (Rowland and Hollar, 2015). However, several participants emphasized the need for time and support to become increasingly familiar with external health literacy materials available on the ward, ensuring they provide adequate and accurate information. Another valuable tool was teach-back, which has been proven beneficial for RNs’ clinical practice (Brown et al., 2025) and was consistently used throughout the intervention. Promoting the use of teach-back to check for understanding and correct misunderstandings is essential for improving communication clarity and alleviating negative consequences of low health literacy (Coleman et al., 2017; Brown et al., 2025).

### 4.2 Strengths and limitations

This study had several strengths and limitations. One of its key strengths was the use of a multimethod evaluation strategy that integrated both quantitative surveys and semi-structured interviews, providing detailed information about the intervention's feasibility, acceptability, usefulness, and relevance. Another strength that we provided is an important lens for practice-based development and field testing of the health literacy nurse role and systematized health literacy sensitivity. The systematic approach to developing the intervention, along with its implementation and evaluation of feasibility, demonstrates how an intervention can be deployed and assessed in real-world clinical settings. The involvement of an advisory panel, which used a comprehensive co-creation approach that engaged multiple key stakeholders and utilized local expertise, further strengthened the intervention’s relevance and applicability. Further, this intervention was based on two previous studies conducted in the same hospital, which explored the experiences of healthcare personnel and the associations between health literacy sensitivity, work-related factors, and mental well-being. This is beneficial because it builds on clinical needs within the same population, ensuring consistency in the research approach. Moreover, transcripts from the qualitative interviews were returned to participants for validation of the results (Birt et al., 2016).

The study has several limitations. Notably, two domains of the Norwegian version of the Instrument of Health Literacy Competencies (Borge et al., 2025), domain 4 (“Design of easy-to-use materials”) and domain 6 (“Checking for understanding”) yielded Cronbach’s α coefficients below 0.70, indicating limited internal consistency (Tavakol & Dennick, 2011). Low reliability reduces statistical power to detect true pre–post changes and attenuates observed effect sizes; thus, these results should be interpreted with caution (Hussey et al., 2025). A further limitation is the potential conflict of interest arising from the data collectors’ employment within the same institution as the participants. This inherent power dynamic may have influenced participant responses and could lead to a skewed representation of the workplace environment (Ayrton, 2024). Moreover, the pre-post design, while useful for feasibility assessment, lacks a control group, limiting the ability to attribute changes solely to the intervention due to potential external influences or temporal trends, and is not a design that evaluates effect, such as a randomized control trial (Polit and Beck, 2012). Furthermore, sample size selection was based on practical feasibility and resource limitations, not a statistical power analysis. Consequently, the study is potentially-underpowered (Montgomery, 2025). Another potential limitation is that all items in the pre-post intervention survey were mandatory, which might have led to non-response bias (Prince, 2012). However, this potential bias was mitigated by the high overall pre-intervention response rate and the attrition analysis indicating no significant differences between survey completers and non-completers. Lastly, cultural factors within the medical ward may have influenced the observed positive changes, as intervention ward employees had strong pre-existing trust, an open communication climate, and a shared professional identity (Marsiglia and Booth, 2015).

### 4.3 Practice implications and further studies

The empirical evidence from this study may be valuable to healthcare leaders in hospitals and primary care settings. The components of the intervention can be tailored to different care environments, and the motivating and guiding role of the health literacy nurse is not limited to medical wards. Providing participants with opportunities for professional development through project groups was experienced as particularly useful for their health literacy sensitivity. However, RNs may require ongoing training and reminders to successfully use communication techniques. Future researchers should include larger samples across hospital departments and in a randomized controlled design, and collaboration among multiple professions may be necessary to evaluate the potential effectiveness of the health literacy intervention in a broader context. Additionally, cost-benefit and efficiency analyses should be conducted.

### 4.4 Conclusion

In this intervention feasibility study, we demonstrated a statistically-significant increase in participants’ awareness and understanding of health literacy sensitivity through routine practices, collaboration, and access to resources, including support from a dedicated health literacy nurse. Health literacy sensitivity remained a balance between rewarding engagement and burdensome responsibility. Participants found the intervention to be feasible and acceptable in its current form. We have demonstrated that it was possible to enhance RNs´ health literacy sensitivity without harmful consequences for work-related factors or diminishing mental well-being. The long-term sustainability of these changes after the intervention period remains uncertain.

## Funding

The Norwegian Nursing Organization and Foundation Dam (grant number SDAM_HEL508707) funded the project in collaboration with the Norwegian non-profit health organization, LHL: the National Association for Heart, Lung, and Stroke.

## CRediT authorship contribution statement

**Malene Nerbøvik Stavdal:** Writing – review & editing, Writing – original draft, Visualization, Validation, Resources, Project administration, Methodology, Investigation, Funding acquisition, Formal analysis, Data curation, Conceptualization. **Marie Hamilton Larsen:** Writing – review & editing, Visualization, Validation, Supervision, Methodology, Conceptualization. **Ingeborg Strømseng Sjetne:** Writing – review & editing, Visualization, Validation, Supervision, Investigation, Conceptualization. **Astrid Klopstad Wahl:** Writing – review & editing, Visualization, Validation, Supervision, Investigation, Conceptualization. **Caryl L. Gay:** Writing – review & editing, Visualization, Validation, Methodology, Conceptualization. **Monica Bukkøy Kjetland:** Writing – review & editing, Funding acquisition, Data curation, Conceptualization. **Anners Lerdal:** Writing – review & editing, Resources, Funding acquisition. **Christine Råheim Borge:** Writing – review & editing, Visualization, Validation, Supervision, Resources, Methodology, Investigation, Funding acquisition, Formal analysis, Data curation, Conceptualization.

## Declaration of competing interest

Malene N Stavdal reports financial support was provided by The Norwegian Nursing Organization and Foundation Dam. If there are other authors, they declare that they have no known competing financial interests or personal relationships that could have appeared to influence the work reported in this paper.

## References

[bib0001] Andersen M.H., Urstad K.H., Larsen M.H., Henrichsen G.F., Engebretsen E., Ødemark J., Stenehjem A.E., Reisæter A.V., Nordlie A., wahealth literacy A.K. (2022). Intervening on health literacy by knowledge translation processes in kidney transplantation: A feasibility study. J. Ren. Care.

[bib0002] Anselmann V., Halder S., Sauer S. (2024). Nursing students’ Health literacy and strategies to foster patients’ Health literacy. Int. J. Env. Res Public Health.

[bib0003] Ayrton R. (2024).

[bib0004] Birt L., Scott S., Cavers D., Campbell C., Walter F. (2016). Member checking: A tool to enhance trustworthiness or merely a nod to validation?. Qual. Health Res..

[bib0005] Borge C.R., Wahl A.K., Larsen M.H., Andersen M.H., Stavdal M.N., Hermansen Å. (2025). Validation of the Instrument of Health Literacy Competencies (IOHLC) in Norwegian interdisciplinary health care professionals in specialist health care service. J. Interprof. Educ. Pract..

[bib0006] Bowen D.J.P., Kreuter M.P.M.P.H., Spring B.P.A., Cofta-Woerpel L.P., Linnan L.S.C., Weiner D.P., Bakken S.R.N.D.F., Kaplan C.P.P., Squiers L.P., Fabrizio C.P., Fernandez M.P. (2009). How we design feasibility studies. Am. J. Prev. Med..

[bib0007] Braun V., Clarke V. (2022).

[bib0008] Brown C., Dotson B., Montgomery J., Sutterfield C., Maharaj G. (2025). Evaluating the effectiveness of using the teach-back method to improve the health literacy of individuals in the community. J. Community Health Nurs..

[bib0009] Bruyneel A., Dello S., Dauvergne J.E., Kohnen D., Sermeus W. (2025). Prevalence and risk factors for burnout, missed nursing care, and intention-to-leave the job among intensive care unit and general ward nurses: A cross-sectional study across six European countries in the COVID-19 era. Intensive Crit. Care Nurs..

[bib0010] Carthon J.M., Hatfield L., Plover C., Dierkes A., Davis L., Hedgeland T., Sanders A.M., Visco F., Holland S., Ballinghoff J., Del Guidice M., Aiken L.H. (2019). Association of Nurse Engagement and Nurse Staffing on Patient Safety. J. Nurs. Care Qual..

[bib0011] Cawthon C., Mion L.C., Willens D.E., Roumie C.L., Kripalani S. (2014). Implementing routine health literacy assessment in hospital and primary care patients. Jt. Comm. j. qual. patient saf..

[bib0012] Cesar F.C.R., Moraes K.L., Brasil V.V., Alves A.G., Barbosa M.A., Oliveira L.M.D.A.C. (2022). Professional Responsiveness to Health literacy: A scoping review. Health Lit. Res. Pr..

[bib0013] Chang L.-C., Chen Y.-C., Liao L.-L., Wu F.L., Hsieh P.-L., Chen H.-J. (2017). Validation of the instrument of health literacy competencies for Chinese-speaking health professionals. PLoS. One.

[bib0014] Cocchieri A., Cristofori E., Nurchis M.C., Nursing, Public Health Group N., Public Health G., Damiani G., Cesare M. (2025). Nursing complexity and health literacy as determinants of patient outcomes: A prospective one-year multicenter cohort study. Nurs. Rep..

[bib0015] Cocchieri A., Pezzullo A.M., Cesare M., De Rinaldis M., Cristofori E., D'agostino F. (2024). Association between health literacy and nursing care in hospital: A retrospective study. J. Clin. Nurs..

[bib0016] Coleman C., Hudson S., Maine L. (2013). Health literacy practices and educational competencies for Health professionals: A consensus study. J. Health Commun..

[bib0017] Coleman C., Hudson S., Pederson B. (2017). Prioritized health literacy and clear communication practices for health care professionals. Health Lit. Res. Pr..

[bib0018] Debello M., Wu T.Y. (2025). A descriptive study on health literacy: knowledge, application, and confidence of student nurses in the U.S. SAGe Open. Nurs..

[bib0019] Djojo A., Suhariyanto S., Yuniar L., Suni A., Riani E., Ervandi Y., Walvri S., Aprizal A., Hariyati R.T.S., Handiyani H. (2020). Effectiveness of an intervention based on Peplau’s model on health literacy among nurses who smoke: A quasi-experimental study. J. Ners. (Surabaya).

[bib0020] Duncan E., O'cathain A., Rousseau N., Croot L., Sworn K., Turner K.M., Yardley L., Hoddinott P. (2020). Guidance for reporting intervention development studies in health research (GUIDED): an evidence-based consensus study. BMJ Open..

[bib0021] Flowers S.-L.D.A., Guillén-Solà M., Sansó N., Galiana L. (2024). Compassionate care: A qualitative exploration of nurses’ Inner resources in the face of burnout. Nurs. rep. (Pavia Italy).

[bib0022] Fossum M., Opsal A., Ehrenberg A. (2022). Nurses' sources of information to inform clinical practice: an integrative review to guide evidence-based practice. Worldviews. Evid. Based. Nurs..

[bib0023] Goeman D., Conway S., Norman R., Morley J., Weerasuriya R., Osborne R.H., Beauchamp A. (2016). Optimising health literacy and access of service provision to community dwelling older people with diabetes receiving home nursing support. J. Diabetes. Res..

[bib0024] Green S., Markaki A., Baird J., Murray P., Edwards R. (2020). Addressing healthcare professional burnout: A quality improvement intervention. Worldviews. Evid. Based. Nurs..

[bib0025] Hoffmann T.C., Glasziou P.P., Boutron I., Milne R., Perera R., Moher D., Altman D.G., Barbour V., Macdonald H., Johnston M., Lamb S.E., Dixon-Woods M., Mcculloch P., Wyatt J.C., Chan A.W., Michie S. (2014). Better reporting of interventions: template for intervention description and replication (TIDieR) checklist and guide. BMJ.

[bib0026] Hussey I., Alsalti T., Bosco F., Elson M., Arslan R. (2025). An aberrant abundance of Cronbach’s alpha values at .70. Adv. Methods Pract. Psychol. Sci..

[bib0027] Jackson K., Bazeley P. (2019).

[bib0028] Jensen N.H., Aaby A., Ryom K., Maindal H.T. (2021). A CHAT about health literacy – a qualitative feasibility study of the Conversational Health Literacy Assessment Tool (CHAT) in a Danish municipal healthcare centre. Scand. J. Caring Sci..

[bib0029] Karlsson A.C., Gunningberg L., Bäckström J., Pöder U. (2019). Registered nurses’ perspectives of work satisfaction, patient safety and intention to stay – A double-edged sword. J. Nurs. Manage.

[bib0030] Karuranga S., Sørensen K., Coleman C., Mahmud A.J (2017). Health literacy competencies for European Health Care personnel. Health Lit Res Pr..

[bib0031] Kerby D.S. (2014). The simple difference formula: an approach to teaching nonparametric Correlation1. Compr. Psychol..

[bib0032] Kroenke, K., Spitzer, R. L. & Williams, J. B. 2003. The patient Health Questionnaire-2: validity of a two-item depression screener. *Med. Care,* 41**,** 1284-92. 10.1097/01.MLR.0000093487.78664.3C 14583691

[bib0033] Kroenke K., Spitzer R.L., Williams J.B., Löwe B. (2007). Anxiety disorders in primary care: prevalence, impairment, comorbidity, and detection. Ann. Intern. Med..

[bib0034] Lake E.T., Gil J., Moronski L., Mchugh M.D., Aiken L.H., Lasater K.B. (2024). Validation of a short form of the practice environment scale of the nursing work index: the PES-5. Res. Nurs. Health.

[bib0035] Larrotta-Castillo D., Moreno-Chaparro J., Amaya-Moreno A., Gaitán-Duarte H., Estrada-Orozco K. (2023). Health literacy interventions in the hospital setting: an overview. Health Promot. Int..

[bib0036] Le C., Finbråten H.S., Pettersen K.S., Joranger P., Guttersrud Ø (2021).

[bib0037] Loan L.A., Parnell T.A., stichealth literacyer J.F., Boyle D.K., Allen P., Vanfosson C.A., Barton A.J. (2018). Call for action: nurses must play a critical role to enhance health literacy. Nurs. Outlook..

[bib0038] MAGNET4EUROPE (2020). https://www.nursing.upenn.edu/live/files/1246-m4egap-analysis-toolinternational#:~:text=Purpose:,38:%20All%20include%20Baccalaureate%20requirements.

[bib0039] Marshall N., Butler M., Lambert V., Timon C.M., Joyce D., Warters A. (2025). Health literacy interventions and health literacy-related outcomes for older adults: a systematic review. BMC Health v Res..

[bib0040] Marsiglia F.F., Booth J.M. (2015). Cultural adaptation of interventions in real practice settings. Res. Soc. Work Pr..

[bib0041] Mccarthy D.M., Waite K.R., Curtis L.M., Engel K.G., Baker D.W., Wolf M.S. (2012). What did the doctor say? Health literacy and recall of medical instructions. Med. Care.

[bib0042] Mccaskill A., Gasch-Gallen A., Montero-Marco J. (2024). The effect of nurse health literacy interventions on patient health literacy scores in specialty consultations: a quasi-experimental study. BMC. Nurs..

[bib0043] Meggetto E., Kent F., Ward B., Keleher H. (2020). Factors influencing implementation of organizational health literacy: a realist review. J. Health Organ. Manage.

[bib0044] Montgomery R. (2025). Sample size justification in feasibility studies: moving beyond published guidance. Pilot. Feasibility. Stud..

[bib0045] Moore G.F., Audrey S., Barker M., Bond L., Bonell C., Hardeman W., Moore L., O’cathain A., Tinati T., Wight D., Baird J. (2015). Process evaluation of complex interventions: Medical Research Council guidance. BMJ.

[bib0046] Moreno-Martínez M., Sánchez-Martínez I. (2025). The associated factors of work engagement, work overload, work satisfaction, and emotional exhaustion and their effect on healthcare workers: A cross-sectional study. Healthc. (Basel).

[bib0047] Nutbeam D. (1998). Health promotion glossary. Health Promot. Int..

[bib0048] Nutbeam D., Muscat D.M. (2020). Advancing health literacy interventions. Stud Health Technol Inf..

[bib0049] O’hara J., Hawkins M., Batterham R., Dodson S., Osborne R.H., Beauchamp A. (2018). Conceptualisation and development of the Conversational Health Literacy Assessment Tool (CHAT). BMC Health v Res.

[bib0050] Pallant J. (2020).

[bib72] Pelizzari N., Covolo L., Ceretti E., Fiammenghi C., Gelatti U. (2025). Defining, assessing, and implementing organizational health literacy: barriers, facilitators, and tools - a systematic review. BMC Health Serv. Res..

[bib0051] Polit D.F., Beck C.T. (2012).

[bib0052] Prince M., WRIGHT P., STERN J., PHELAN M. (2012). Core Psychiatry.

[bib0053] Rowland J., Hollar D. (2015). Promoting health literacy for people with disabilities and clinicians through a teamwork model. J. Fam. Strengths.

[bib0054] Schaufeli W., Shimazu A., Hakanen J., Salanova M., De Witte H. (2017). An ultra-short measure for work engagement: the UWES-3 validation across five countries. Eur. J. Psychol. Assess..

[bib0055] Sermeus W., Aiken L.H., Ball J., Bridges J., Bruyneel L., Busse R., De Witte H., Dello S., Drennan J., Eriksson L.E., Griffiths P., Kohnen D., Köppen J., Lindqvist R., Maier C.B., Mchugh M.D., Mckee M., Rafferty A.M., Schaufeli W.B., Sloane D.M., Alenius L.S., Smith H., MAGNET4EUROPE-CONSORTIUM (2022). A workplace organisational intervention to improve hospital nurses’ and physicians’ mental health: study protocol for the Magnet4Europe wait list cluster randomised controlled trial. BMJ Open..

[bib0056] Skivington K., Matthews L., Simpson S.A., Craig P., Baird J., Blazeby J.M., Boyd K.A., Craig N., French D.P., Mcintosh E., Petticrew M., Rycroft-Malone J., White M., Moore L. (2021). A new framework for developing and evaluating complex interventions: update of Medical Research Council guidance. BMJ.

[bib0057] Sørensen K., Brand H. (2014). Health literacy lost in translations? Introducing the European Health Literacy Glossary. Health Promot. Int..

[bib0058] Sørensen K., Levin-Zamir D., Duong T.V., Okan O., Brasil V.V., Nutbeam D. (2021). Building health literacy system capacity: a framework for health literate systems. Health Promot. Int..

[bib0059] Stavdal M., Hermansen Å., Sjetne I., Larsen M., wahealth literacy A., Kohnen D., Gay C., Lerdal A., Borge C. (2025). Associations between work-related factors, mental well-being, and health literacy sensitivity: A cross-sectional study among healthcare personnel. J. Healthc. Leadersh..

[bib0060] Stavdal M.N., Larsen M.H., wahealth literacy A.K., Sjetne I.S., Lerdal A., Gay C.L., Borge C.R. (2025). Healthcare personnel experiences with health literacy sensitivity in relation to work satisfaction and stress: A qualitative study. J. Multidiscip. Heal..

[bib0061] Tavakol M., Dennick R. (2011). Making sense of Cronbach's alpha. Int. J. Med. Educ..

[bib0062] Trezona A., Dodson S., Osborne R.H. (2018). Development of the Organisational Health Literacy Responsiveness (Org-health literacyR) self-assessment tool and process. BMC Health v Res.

[bib0063] tsichealth literacya L., Patelarou E., Detorakis E., Tsilimbaris M.K., Patelarou A.E., Giakoumidakis K. (2025). Enhancing health literacy and self-management in Glaucoma patients: evidence from a nurse-led educational intervention. Healthc. (Basel).

[bib0064] Volandes A.E., Paasche-Orlow M.K. (2007). Health literacy, Health inequality and a just healthcare system. Am. J. Bioeth..

[bib0065] Walters R., Leslie S.J., Polson R., Cusack T., Gorely T. (2020). Establishing the efficacy of interventions to improve health literacy and health behaviours: a systematic review. BMC. Public Health.

[bib0066] Wang K.Y., Chou C.C., Lai J.C.Y (2019). A structural model of total quality management, work values, job satisfaction and patient-safety-culture attitude among nurses. J. Nurs. Manage.

[bib0067] WORLD HEALTH ORGANIZATION (n.d.). Health literacy. Ninth Global Conference on Health Promotion. https://www.who.int/teams/health-promotion/enhanced-wellbeing/ninth-global-conference/health-literacy.

[bib0068] Wilandika A., Pandin M.G.R., Yusuf A. (2023). The roles of nurses in supporting health literacy: a scoping review. Front. Public Health.

[bib0069] Wittenberg E., Ferrell B., Kanter E., Buller H. (2018). Health literacy: exploring nursing challenges to providing support and understanding. Clin. J. Oncol. Nurs..

[bib0070] Wynia M.K., Osborn C.Y. (2010). Health literacy and communication quality in Health Care Organizations. J. Health Commun..

[bib0071] Zhang J., Rehman S., Addas A., Ahmad J. (2024). Influence of work-life balance on mental health among nurses: the mediating role of psychological capital and job satisfaction. Psychol. Res. Behav. Manage.

